# An updated checklist of ants (Hymenoptera, Formicidae) of Bulgaria, after 130 years of research

**DOI:** 10.3897/BDJ.10.e95599

**Published:** 2022-11-09

**Authors:** Albena Lapeva-Gjonova, Vera Antonova

**Affiliations:** 1 Sofia University, Sofia, Bulgaria Sofia University Sofia Bulgaria; 2 Bulgarian Academy of Sciences, Sofia, Bulgaria Bulgarian Academy of Sciences Sofia Bulgaria

**Keywords:** the Balkans, conservation, endemic species, exotic species, inventory, myrmecofauna

## Abstract

**Background:**

The Bulgarian myrmecofauna is one of the richest in the Balkans. This is a result of both the physicogeographical and paleoecological features of the area, as well as relatively well-studied fauna. The earliest myrmecological paper on Bulgarian fauna, listing 54 species, was published 130 years ago. The publication was later followed by numerous new faunistic records and three comprehensive reviews that significantly widened knowledge on the ant diversity from this country. The most recent checklist was released 12 years ago and considered 163 ant species from 40 genera.

**New information:**

This work provides an updated checklist of 195 ant species from 43 genera occurring in Bulgaria. Since the last Bulgarian catalogue of ants, 44 species have been added, while 24 species have been synonymised or excluded after critical analysis of the last taxonomic revisions. Additionally, we discuss the status and distribution of 12 species described from Bulgaria, 23 species considered endemic and subendemic for this country, 19 species with conservation status and four non-native species.

## Introduction

Bulgaria is amongst the Balkan countries with the richest ant fauna. There are several factors that favour the existence of more than 190 ant species. The country is located in the south-eastern part of the Balkan Peninsula, considered as an important hotspot of biodiversity in Europe, with 96 types of habitats referring to three biogeographical regions – Black Sea, Continental and Alpine (European Environment Agency 2022). The Balkans act as a connecting corridor between Europe and Asia. Due to its geographic location and paleoecological events, two major zoogeographical complexes can be distinguished – northern (Holarctic-Eurosiberian) complex of cold-tolerant species and southern (Mediterranean-Central Asian) complex of thermophilic species (Hubenov 2008). The latter one includes a limited number of steppe elements (NW and NE Bulgaria), Anatolian and Iranian migrants (SE Bulgaria) and Pontian elements (eastern Bulgaria). In addition, the Bulgarian fauna includes a number of endemic and subendemic species and few exotic species. The high number of ant species (in comparison with other Balkan countries) is also due to the numerous studies on the Bulgarian myrmecofauna conducted in the last decade (see below).

The earliest paper on the myrmecofauna of Bulgaria was published 130 years ago, when Auguste Forel (1848–1931), a Swiss myrmecologist, recorded 54 ant species from various regions of the country and described three species as new to science (Forel 1892). Later, three other comprehensive reviews of the ant fauna in Bulgaria, made by Agosti and Collingwood (1987), Atanassov and Dlusskij (1992) and Lapeva-Gjonova et al. (2010), enriched knowledge on biodiversity of this country. In the list of the Balkan ants, Agosti and Collingwood (1987), based on literature and collection data, reported 112 species for Bulgaria. Exactly 100 years after the publication of the first paper on the ants of Bulgaria, Atanassov and Dlusskij (1992) presented data on the taxonomy, distribution and ecology of 111 ant species from 36 genera and four subfamilies, with identification keys to all taxa. The most recent review of 163 ant species from 40 genera (Lapeva-Gjonova et al. 2010) was prepared mainly based on published records and updated taxonomic status of taxa listed in papers preceding its publication.

Since the publication of the most recent catalogue, 44 more species have been added to the list. Some of them are new faunistic findings for the country, while others are new species mentioned for Bulgaria in taxonomic works covering also the Balkan myrmecofauna (e.g. Seifert 2012, Csősz et al. 2014, Csősz et al. 2015, Seifert and Csősz 2015, Seifert 2016, Seifert and Galkowski 2016, Wagner et al. 2017, Csősz et al. 2018, Steiner et al. 2018, Bračko et al. 2019, Seifert 2020). The high species diversity in the Balkan Peninsula is of considerable importance and has great conservation value as recognised by its hotspot status (Hewitt 2011). In recent years, the most significant progress in the study of the Balkan ant fauna has been made on Greek ants. Data on over 300 species (Salata and Borowiec 2018a), including their distribution and ecology, have been established. Additionally, a number of taxonomic revisions on specific groups of species and genera have been carried out. Important additions to the regional ant fauna of the Balkans were also made for Slovenia, Montenegro and the Republic of North Macedonia (Bračko et al. 2014a, Bračko et al. 2014b, Bračko et al. 2016).

The updated list of Bulgarian ants in the present study brings together the scattered information from numerous taxonomic and faunistic publications, justifies exclusion of some dubious and erroneous records and highlights the importance of such inventories for assessment and conservation of biological diversity.

## Materials and methods

The current checklist is based on the available taxonomic and faunistic literature concerning the Bulgarian myrmecofauna. Publications since the last Bulgarian catalogue of ants (Lapeva-Gjonova et al. 2010) till recently are considered. We make critical reviews on the taxonomic data on some species.

The genera in the list are arranged by subfamilies and tribes. The species are listed alphabetically and by subgenera (if available) as their actual names are generally agreed with the Online catalogue of the ants of the world by Bolton (2022) and the most recent publications. The changes in taxon names proposed by Ward et al. (2015) for social parasitic genera *Anergates*, *Chalepoxenus*, *Myrmoxenus* and *Teleutomyrmex* were not taken into account, based on ongoing discussions and arguments to maintain stability in names (Seifert et al. 2016, Kiran et al. 2017). The excluded species from the last catalogue and subsequent articles are justified by relevant studies. The following abbreviations for the conservation status according to the IUCN Red List of Threatened Species (IUCN 2022), if any, have been used: Vulnerable (VU), Near Threatened (NT), Lower Risk (LR), Least Concern (LC) and Bulgarian Biodiversity Act (BBA). In the Notes section after the current species name, only the very first report for Bulgaria is given and if the species is endemic or subendemic.

## Checklists

### Checklist of the ants of Bulgaria

#### Amblyoponinae

6DB4491B-6345-5546-BE8C-9F62A2F9860D

#### Amblyoponini

51140EBF-8C0C-58E2-8E3C-6037697EE181

#### Stigmatomma denticulatum

Roger, 1859

0933FB72-4E9A-54A1-9191-B7EF88E65603

##### Notes

Forel (1892)

#### Stigmatomma impressifrons

Emery, 1869

A4E6D3CB-0721-5E29-962B-9F35576DEF70

##### Notes

Atanassov and Dlusskij (1992)

#### Dolichoderinae

01AF98FB-3457-5737-AB0E-E5F3B723F71D

#### Bothriomyrmecini

4EE64740-1466-5756-AA3E-898F0AA51C7C

#### Bothriomyrmex communista

Santschi, 1919

28B740BD-C898-59C6-BABC-D842F030F822

##### Notes

Atanassov (1964)

#### Bothriomyrmex corsicus

Santschi, 1923

7A92DCD0-6264-5316-8974-6916D9653EF6

##### Notes

Vassilev (1984)

#### Dolichoderini

23B6513A-1023-509A-ABBA-5084541C1E0F

#### Dolichoderus quadripunctatus

(Linnaeus, 1771)

5A766B8F-8278-54ED-92BB-BDBFD4896B82

##### Notes

Forel (1892)

#### Leptomyrmecini

C34B6FE4-E18A-5929-A8CE-F683A8438BC3

#### Linepithema humile

(Mayr, 1868)

F90E122A-080E-5CB3-9A53-71A25FD47ACF

##### Notes

Atanassov and Dlusskij (1992)

#### Tapinomini

E6EBF211-E0A9-5CA5-A1BA-7736940A5282

#### Liometopum microcephalum

(Panzer, 1798)

964E4B7F-1EDD-53B5-AF49-6A22C9A8BABE

##### Notes

Forel (1892)

#### Tapinoma erraticum

(Latreille, 1798)

A4EC0552-5257-5273-ADED-FC27AF890F57

##### Notes

Forel (1892)

#### Tapinoma subboreale

Seifert, 2012

8F0AC8C8-89EF-5A2C-8EA7-2A2BFE4FCBE0

##### Notes

Forel (1892)

#### Formicinae

42AD20BA-8709-522B-B4A7-E5D257B8801A

#### Camponotini

A3355A8B-444F-56E1-9377-80284A5DF55E

#### Camponotus (Camponotus) herculeanus

(Linnaeus, 1758)

9DE50819-9E3A-5589-8C19-F3B8E47382B8

##### Notes

Forel (1892)

#### Camponotus (Camponotus) ligniperda

(Latreille, 1802)

9BD2E008-2449-59E3-AC94-7E86F7C1C4C8

##### Notes

Forel (1892)

#### Camponotus (Camponotus) vagus

(Scopoli, 1763)

ADC3E140-F592-5E02-A792-A9A3B7C3AD22

##### Notes

Forel (1892)

#### Camponotus (Myrmentoma) aegaeus

Emery, 1915

CAF8F35B-9466-5D9D-85B2-CB4ED7021E31

##### Notes

Lapeva-Gjonova (2011); a Balkan-Anatolian subendemic.

#### Camponotus (Myrmentoma) atricolor

(Nylander, 1849)

FC281028-E781-5FB4-B5F1-0ED0A0794301

##### Notes

Forel (1892)

#### Camponotus (Myrmentoma) dalmaticus

(Nylander, 1849)

C64BA33E-AC34-593D-B048-0045E912B011

##### Notes

Forel (1892)

#### Camponotus (Myrmentoma) fallax

(Nylander, 1856)

DD19133E-1F22-5D8B-8DB2-DA9AB83B72AF

##### Notes

Atanassov (1934)

#### Camponotus (Myrmentoma) gestroi

Emery, 1878

60C185C0-05A4-588E-BD5C-7706BB4EDFB5

##### Notes

Lapeva-Gjonova (2011)

#### Camponotus (Myrmentoma) lateralis

(Olivier, 1792)

D54BFE63-C9A7-5DB6-A1C2-2472A1032200

##### Notes

Forel (1892)

#### Camponotus (Myrmentoma) piceus

(Leach, 1825)

0B2DDCBF-8884-5C02-96EE-E37440DA9E5D

##### Notes

Forel (1892)

#### Camponotus (Myrmentoma) tergestinus

Müller, 1921

516E600B-FB58-5295-B3BE-13EB26E49AD0

##### Notes

Lapeva-Gjonova and Kiran (2012)

#### Camponotus (Tanaemyrmex) aethiops

(Latreille, 1798)

B84E7E63-4F91-51DE-AF71-0DA982C323E6

##### Notes

Forel (1892)

#### Camponotus (Tanaemyrmex) ionius

Emery, 1920

953EB84F-4EAB-5759-905D-8E1A1395D5B2

##### Notes

Atanassov (1964); a Balkan-Anatolian subendemic.

#### Camponotus (Tanaemyrmex) oertzeni

Forel, 1889

3EFFE54F-7119-5EDE-953F-B082EE56FCB7

##### Notes

Lapeva-Gjonova and Santamaria (2011)

#### Camponotus (Tanaemyrmex) samius

Forel, 1889

94A81089-A31A-55AF-91C7-BE1D9F601A8D

##### Notes

Atanassov (1964); a Balkan-Anatolian subendemic.

#### Camponotus (Tanaemyrmex) universitatis

Forel, 1890

F734F1B9-CF3C-581B-A06A-6DAB95659B00

##### Conservation status

Vu D2

##### Notes

Lapeva-Gjonova and Kiran (2012)

#### Colobopsis truncata

(Spinola, 1808)

49ACA70E-C866-5A98-BC58-C5D94EBB2207

##### Notes

Forel (1892)

#### Formicini

C0F6949B-11B2-5F6E-A394-01AF3C31C4A5

#### Cataglyphis aenescens

(Nylander, 1849)

1AFBFB6A-55A0-5363-B84D-86F7A974B2B2

##### Notes

Forel (1892)

#### Cataglyphis nodus

(Brullé, 1833)

432F56DF-C060-5668-882F-0379EF5A30B9

##### Notes

Forel (1892)

#### Cataglyphis viaticoides

(André, 1881)

BBF2C02E-DEC7-59E7-8EAB-BA7184945667

##### Notes

Atanassov (1982)

#### Formica (Coptoformica) exsecta

Nylander, 1846

C59A00C9-FDFC-562D-9DF1-6C8CE8724AA1

##### Notes

Emery (1914)

#### Formica (Coptoformica) pressilabris

Nylander, 1846

0861B3AB-FD05-5CDF-91C5-070981BC79B5

##### Notes

Atanassov (1934)

#### Formica (Formica) aquilonia

Yarrow, 1955

A3F165C8-9566-5E6F-AF4D-F35B94C30F18

##### Conservation status

LR/NT, Corine (Annex 4)

##### Notes

Wesselinoff (1973)

#### Formica (Formica) lugubris

Zetterstedt, 1838

A2620820-5B59-55AD-AEC4-06CD86532F6B

##### Conservation status

LR/NT, Corine (Annex 4)

##### Notes

Otto et al. (1962)

#### Formica (Formica) polyctena

Förster, 1850

3A9B8293-A964-5C51-8E43-E983D33B7384

##### Conservation status

LR/NT, Corine (Annex 4)

##### Notes

Wesselinoff (1973)

#### Formica (Formica) pratensis

Retzius, 1783

CFC0D400-0DE7-5751-8D2C-D27106B2F8D9

##### Conservation status

LR/NT, Corine (Annex 4)

##### Notes

Forel (1892)

#### Formica (Formica) rufa

Linnaeus, 1761

0A11A700-F580-594A-BC21-A2537867B22A

##### Conservation status

LR/NT, Corine (Annex 4), BBA (2002) Annexes 2 and 3

##### Notes

Forel (1892)

#### Formica (Formica) truncorum

Fabricius, 1804

7FEEB64F-1433-5407-B94C-78B98AB10564

##### Conservation status

Corine (Annex 4)

##### Notes

Wesselinoff (1973)

#### Formica (Raptiformica) sanguinea

Latreille, 1798

BE739067-462F-59B6-BEF8-5A094F73AE30

##### Notes

Forel (1892)

#### Formica (Serviformica) cinerea

Mayr, 1853

931E9440-060E-5CE9-B405-C3959B557BC9

##### Notes

Forel (1892)

#### Formica (Serviformica) clara

Forel, 1886

97DF924C-C357-53CF-BD2F-3D55231C7588

##### Notes

Barrett (1970)

#### Formica (Serviformica) cunicularia

Latreille, 1798

4410E284-E773-562C-BE86-5F67A4B097A7

##### Notes

Forel (1892)

#### Formica (Serviformica) fusca

Linnaeus, 1758

3AD62921-BAAE-5C88-905E-42FFE1456D8F

##### Notes

Forel (1892)

#### Formica (Serviformica) gagates

Latreille, 1798

BA5DDC8C-B2FC-5372-9DEB-E71785941E6A

##### Notes

Forel (1892)

#### Formica (Serviformica) lemani

Bondroit, 1917

62A4517A-3232-54E1-A538-36F315903628

##### Notes

Atanassov (1936)

#### Formica (Serviformica) picea

Nylander, 1846

628F91AD-6F03-589B-A104-CC960C673228

##### Notes

Atanassov and Dlusskij (1992)

#### Formica (Serviformica) glauca

Ruzsky, 1896

4BAB78E5-28EF-5532-A95A-B0ADA2352445

##### Notes

Atanassov and Vasileva (1976)

#### Formica (Serviformica) rufibarbis

Fabricius, 1793

BE33BDD4-D413-5DF2-AD68-ED46A71814AF

##### Notes

Forel (1892)

#### Polyergus rufescens

(Latreille, 1798)

76FFA3BE-DBE1-569E-BC27-69F76E44CA59

##### Notes

Forel (1892)

#### Proformica kobachidzei

K. Arnoldi, 1968

3FA439A2-0D4F-5753-AF65-562D0C323131

##### Notes

Atanassov and Dlusskij (1992); a Ponto-Caucasian subendemic.

#### Proformica korbi

(Emery, 1909)

C9E074A7-AE59-547C-BEEC-ACCF66634C31

##### Notes

Dlussky (1969); a Balkan-Anatolian subendemic.

#### Proformica pilosiscapa

Dlussky, 1969

B85F8F07-A6A8-53A6-98C8-AE3E15DF7393

##### Notes

Dlussky (1969), paratype locality

#### Proformica striaticeps

(Forel, 1911)

2CC62B81-40A6-53E4-80D7-8A77A4628E90

##### Notes

Forel (1892); a Balkan-Anatolian subendemic.

#### Lasiini

80930B3E-B64C-5791-9506-A83CDF0B86E6

#### Lasius (Austrolasius) carniolicus

Mayr, 1861

A3C27898-673A-5729-AB10-7F0C44E1C5ED

##### Notes

Atanassov and Dlusskij (1992)

#### Lasius (Austrolasius) reginae

Faber, 1967

AC3AD596-6F39-5F83-87D6-102D5334FBF3

##### Conservation status

Vu A2c

##### Notes

Lapeva-Gjonova and Borowiec (2022)

#### Lasius (Cautolasius) flavus

(Fabricius, 1782)

B8D61851-B5C1-5975-9115-804106553F13

##### Notes

Atanassov (1934)

#### Lasius (Cautolasius) myops

Forel, 1894

B082582E-5FA3-57C4-AA7C-D30BEAC44892

##### Notes

Atanassov (1952)

#### Lasius (Chthonolasius) balcanicus

Seifert, 1988

8488BA27-DFFF-5069-BCA9-D76CE0ADDAE5

##### Notes

Seifert (1988a), type locality

#### Lasius (Chthonolasius) bicornis

(Förster, 1850)

177BBA85-3ACF-58B7-A6BE-4D379C1ABF0D

##### Notes

Atanassov (1964)

#### Lasius (Chthonolasius) citrinus

Emery, 1922

F85BA8F8-32FE-5A58-8C5C-6BF7C9095E74

##### Notes

Seifert (1988a)

#### Lasius (Chthonolasius) distinguendus

(Emery, 1916)

18F426C8-2216-5AC8-8CFC-4D52A15CA155

##### Notes

Vassilev (1984)

#### Lasius (Chthonolasius) jensi

Seifert, 1982

76294274-A8FC-54B8-B4F8-5DEC8E7AC013

##### Notes

Seifert (1988a)

#### Lasius (Chthonolasius) meridionalis

(Bondroit, 1920)

7D9A9144-90C5-5588-B708-02E875F31CA4

##### Notes

Agosti and Collingwood (1987)

#### Lasius (Chthonolasius) mixtus

(Nylander, 1846)

D49476BF-C77A-5D8F-B877-7ABB65EF9827

##### Notes

Emery (1914)

#### Lasius (Chthonolasius) nitidigaster

Seifert, 1996

E632A49A-849A-5691-A3AD-5A7ADB38AD7F

##### Notes

Agosti and Collingwood (1987) (as *L.rabaudi*), type locality

#### Lasius (Chthonolasius) umbratus

(Nylander, 1846)

957BDC12-AB28-5C45-9C47-5530EE62527D

##### Notes

Forel (1892)

#### Lasius (Dendrolasius) fuliginosus

(Latreille, 1798)

00A11A08-61B5-5363-A37B-9316EAC5E712

##### Notes

Forel (1892)

#### Lasius (Lasius) alienus

(Förster, 1850)

2B365386-61EF-507B-AA45-550FB44CA259

##### Notes

Forel (1892)

#### Lasius (Lasius) bombycina

Seifert & Galkowski, 2016

5A1AFC84-593A-5D10-BCED-E1FDAA090A7B

##### Notes

Seifert and Galkowski (2016)

#### Lasius (Lasius) brunneus

(Latreille, 1798)

F255CB3B-9477-5090-A9B3-72DADAA92F2E

##### Notes

Forel (1892)

#### Lasius (Lasius) emarginatus

(Olivier, 1792)

59038136-D1FB-5D39-987B-27CFA2D32571

##### Notes

Atanassov (1964)

#### Lasius (Lasius) illyricus

Zimmermann, 1935

C1CE40CF-8241-5BBA-A849-A638567369BC

##### Notes

Seifert (2000)

#### Lasius (Lasius) neglectus

Van Loon, Boomsma & Andrasfalvy, 1990

233E7F2E-42E9-5E49-925E-21109447F5C5

##### Notes

Seifert (1992)

#### Lasius (Lasius) niger

(Linnaeus, 1758)

ADCCA8B3-65B9-5EBA-8C09-ED9FFC98CA9D

##### Notes

Forel (1892)

#### Lasius (Lasius) paralienus

Seifert, 1992

D3895056-12B3-5300-B121-D75F9BE31785

##### Notes

Seifert (1992)

#### Lasius (Lasius) platythorax

Seifert, 1991

7B8137B7-8AFE-5BA7-83E6-1D419ABAF611

##### Notes

Antonova and Penev (2006)

#### Lasius (Lasius) psammophilus

Seifert, 1992

8694B7AB-FED5-54A2-95C4-6B5C330341FC

##### Notes

Antonova and Penev (2006)

#### Plagiolepidini

16DDE47D-236E-5C67-B6C7-87EFFD5A6895

#### Lepisiota frauenfeldi

(Mayr, 1855)

32F332F3-1562-576F-BED3-D98373C8BD22

##### Notes

Atanassov (1936)

#### Lepisiota nigra

(Dalla Torre, 1893)

33A4FC2E-7F34-5B2E-8E5D-B0B34E474064

##### Notes

Agosti and Collingwood (1987)

#### Plagiolepis pallescens

Forel, 1889

5621F5AB-99A6-5364-B6EF-E910C7B6B4AD

##### Notes

Atanassov (1964)

#### Plagiolepis pygmaea

(Latreille, 1798)

3E885FF7-ED91-50AA-88B8-1B1E6F60E2BC

##### Notes

Forel (1892)

#### Plagiolepis xene

Stärcke, 1936

42FCB691-A06A-587E-88B8-9C64F6B79B12

##### Notes

Lapeva-Gjonova and Borowiec (2022)

#### Prenolepis nitens

(Mayr, 1853)

03E9F84A-2D86-5EAD-802A-54ED9368CD02

##### Notes

Atanassov (1936)

#### Myrmicinae

98D0CBBD-E786-5473-B693-274C86701356

#### Attini

A19850FE-5535-5413-A1D9-3EE5B0FDF2FE

#### Pheidole balcanica

Seifert, 2016

B128B90E-D53E-54F6-8C09-32876009DF7F

##### Notes

Seifert (2016); a Balkan-Anatolian subendemic.

#### Pheidole pallidula

(Nylander, 1849)

BF22D45B-2FA2-51E9-B7E2-AAC974B06F47

##### Notes

Forel (1892)

#### Strumigenys argiola

(Emery, 1869)

50D50F98-4F45-5577-A8BD-768ECAD10BB8

##### Notes

Lapeva-Gjonova and Ljubomirov (2020)

#### Strumigenys baudueri

(Emery, 1875)

E8BE43C9-C29B-58EB-995C-CF5F74E93BD0

##### Notes

Bezděčka and Bezděčková (2009)

#### Strumigenys tenuipilis

Emery, 1915

4B110A44-35D6-5635-A567-FDC3994BC578

##### Notes

Lapeva-Gjonova and Ljubomirov (2020)

#### Crematogastrini

7405462B-D5F7-52A7-BE66-F29BA22972EB

#### Anergates atratulus

(Schenck, 1852)

7E56AE63-1CF4-5520-9EF5-2CA2E4B5DDA0

##### Conservation status

Vu D2

##### Notes

Atanassov and Dlusskij (1992)

#### Cardiocondyla bulgarica

Forel, 1892

9747311D-E530-5914-AF1C-8EDA398CF070

##### Notes

Forel (1892), type locality; a Balkan-Anatolian subendemic.

#### Cardiocondyla dalmatica

Soudek, 1925

9A392DDA-BCD4-5DB2-A02F-C8A96F7D51F8

##### Notes

Seifert (2003); a Balkan endemic.

#### Cardiocondyla nigra

Forel, 1905

AA3CF107-2F0D-5D64-ACAE-44F24CFC055F

##### Notes

Agosti and Collingwood (1987)

#### Cardiocondyla stambuloffii

Forel, 1892

F32E1094-3EB7-5B3D-B99C-14A54BD386AA

##### Notes

Forel (1892), type locality

#### Chalepoxenus muellerianus

(Finzi, 1922)

1950BA52-2ACB-546E-B540-E0B05F7B67C1

##### Conservation status

Vu D2

##### Notes

Buschinger and Douwes (1993)

#### Crematogaster gordani

Karaman, 2008

45F431EC-2C0C-5B84-895E-51405C8728D9

##### Notes

Borowiec (2014); a Balkan endemic.

#### Crematogaster ionia

Forel, 1911

63186CB0-76B3-549C-AE4C-42386B17F780

##### Notes

Lapeva-Gjonova and Borowiec (2022)

#### Crematogaster lorteti

Forel, 1910

22709D43-2008-552E-8427-55D6001A2427

##### Notes

Lapeva-Gjonova (2011)

#### Crematogaster schmidti

(Mayr, 1853)

8853FDFA-C315-58D5-A95C-07EBFDF2F538

##### Notes

Forel (1892)

#### Crematogaster scutellaris

(Olivier, 1792)

D17EF15C-966E-5E6B-B22C-9F13CFF6882C

##### Notes

Agosti and Collingwood (1987)

#### Crematogaster sordidula

(Nylander, 1849)

8E706D17-BF6B-532A-9C80-FF9A66E9893E

##### Notes

Forel (1892)

#### Formicoxenus nitidulus

(Nylander, 1846)

1CA99DFD-6026-5408-9651-984771C4D224

##### Conservation status

Vu A2c

##### Notes

Atanassov (1936)

#### Harpagoxenus sublaevis

(Nylander, 1849)

2CD33D85-A787-506D-B84F-837A1AC2FE5D

##### Conservation status

Vu A2c

##### Notes

Antonova (2009)

#### Leptothorax acervorum

(Fabricius, 1793)

5608C2B0-54BF-5592-AD3C-76A2442972E9

##### Notes

Forel (1892)

#### Leptothorax muscorum

(Nylander, 1846)

72F95D44-1BDF-5A3B-AB53-D19C8DED6C6B

##### Notes

Atanassov (1952)

#### Myrmecina graminicola

(Latreille, 1802)

04890791-800A-5671-91D4-47F71D40176F

##### Notes

Forel (1895)

#### Myrmoxenus gordiagini

Ruzsky, 1902

C838A5F6-30EB-510A-8D9B-705BA795E606

##### Conservation status

Vu D2

##### Notes

Buschinger and Douwes (1993)

#### Myrmoxenus kraussei

(Emery, 1915)

645A2E9F-1ABC-550B-B5B7-744CFE95C796

##### Conservation status

Vu D2

##### Notes

Ljubomirov (2019)

#### Myrmoxenus ravouxi

(André, 1896)

2860FAF7-A165-5FEF-AB55-CFFE9B8F5E46

##### Conservation status

Vu D2

##### Notes

Buschinger and Douwes (1993)

#### Strongylognathus afer

Emery, 1884

82923DE5-3086-5E17-83C5-EA163F3E93A1

##### Conservation status

Vu D2

##### Notes

Lapeva-Gjonova and Radchenko (2021)

#### Strongylognathus bulgaricus

Pisarski, 1966

E9D5E92E-DC82-517E-97D6-688E5D85B4D1

##### Notes

Viehmeyer (1922), type locality; a Bulgarian endemic.

#### Strongylognathus huberi dalmaticus

Baroni Urbani, 1969

A5A66637-E8F0-5256-BC1D-7A5451A79C9B

##### Notes

Lapeva-Gjonova and Radchenko (2021)

#### Strongylognathus italicus

Finzi, 1924

C8675CA2-A7D0-5F8D-ABC8-22E32FC7B83D

##### Conservation status

Vu D2

##### Notes

Lapeva-Gjonova and Radchenko (2021)

#### Strongylognathus karawajewi

Pisarski, 1966

81E38F4B-AF27-51C1-ACD5-505AB2868DE0

##### Conservation status

Vu D2

##### Notes

Lapeva-Gjonova and Radchenko (2021)

#### Strongylognathus testaceus

(Schenck, 1852)

645534B7-DEAA-5211-99E2-E678EFAB64F1

##### Notes

Atanassov (1964)

#### Teleutomyrmex buschingeri

Lapeva-Gjonova, 2017

EE1D4222-DC4E-5697-9CE0-D2429062B7AE

##### Notes

Kiran et al. (2017), type locality; a Bulgarian endemic.

#### Temnothorax aeolius

(Forel, 1911)

5FD6AC84-B0B7-55E1-B473-96613B7C7479

##### Notes

Lapeva-Gjonova and Borowiec (2022)

#### Temnothorax affinis

(Mayr, 1855)

3DB25946-52C1-5378-92A1-021FB362B315

##### Notes

Forel (1892)

#### Temnothorax bulgaricus

(Forel, 1892)

ED68E8BF-6CA1-5450-ABF8-CDBDF5F526F0

##### Notes

Forel (1892)

#### Temnothorax cf. exilis

(Emery, 1869)

C2C15413-F51D-56C6-B78D-3801E16ACCC0

##### Notes

Lapeva-Gjonova and Borowiec (2022)

#### Temnothorax cf. korbi

(Emery, 1924)

46D5B204-6EEA-5276-ACEA-A95C1D943711

##### Notes

Lapeva-Gjonova et al. (2010)

#### Temnothorax clypeatus

(Mayr, 1853)

E9276030-BE78-5086-8DC4-18ED01BE2EA4

##### Notes

Atanassov (1964)

#### Temnothorax corticalis

(Schenck, 1852)

C2FE450D-4BA5-5C85-8AF4-23B655C2041C

##### Notes

Atanassov (1964)

#### Temnothorax crasecundus

(Seifert & Csősz, 2015)

10836847-8F25-545A-8B79-C0DD702EEC3F

##### Notes

Seifert and Csősz (2015)

#### Temnothorax crassispinus

(Karavaiev, 1926)

D4C1AE9E-830D-5E18-BC36-09D6DF320DE9

##### Notes

Seifert (1995)

#### Temnothorax finzii

(Menozzi, 1925)

8E84B923-872C-524D-B739-05DC93941833

##### Notes

Lapeva-Gjonova and Borowiec (2022)

#### Temnothorax flavicornis

(Emery, 1870)

17851B8A-597A-5F23-96F6-8508141C5236

##### Notes

Lapeva-Gjonova et al. (2014)

#### Temnothorax graecus

(Forel, 1911)

BBD065BD-36BD-52DB-8A4E-A6F76E05394B

##### Notes

Lapeva-Gjonova et al. (2010); a Balkan-Anatolian subendemic.

#### Temnothorax helenae

Csősz, Heinze & Mikó, 2015

AD32360D-C6DC-53E8-9B8B-DB93197ACD91

##### Notes

Csősz et al. (2015); a Balkan endemic.

#### Temnothorax interruptus

(Schenck, 1852)

17B0FB17-5A7B-569B-9932-3AC888A13104

##### Notes

Atanassov and Dlusskij (1992)

#### Temnothorax lichtensteini

(Bondroit, 1918)

4FEBC8DB-B900-5DDB-A358-B13ED1A62086

##### Notes

Csősz et al. (2014)

#### Temnothorax nadigi

(Kutter, 1925)

9D0BF4E1-85EF-5F50-AD6F-28FA52F27D21

##### Notes

Czechowska et al. (1998)

#### Temnothorax nigriceps

(Mayr, 1855)

F30D92B8-F0DC-5B3F-8FEA-EF3AC6A321B7

##### Notes

Agosti and Collingwood (1987)

#### Temnothorax parvulus

(Schenck, 1852)

B2F241CA-1E36-53B0-AC0E-EDF1E53BE62E

##### Notes

Forel (1892)

#### Temnothorax recedens

(Nylander, 1856)

21768776-0C87-511C-9D88-4BD19A278BD9

##### Conservation status

LR/LC

##### Notes

Forel (1892)

#### Temnothorax rogeri

Emery, 1869

E691AE37-8E04-5B73-B37B-797E308830A6

##### Notes

Lapeva-Gjonova and Borowiec (2022); a Balkan endemic.

#### Temnothorax semiruber

(André, 1881)

75A2A8DF-BDAD-5CCE-BCBB-AFB224BEC5D3

##### Notes

Forel (1892)

#### Temnothorax sordidulus

(Müller, 1923)

0D05F0A1-2DFC-5417-BCD1-245FBB8E51D3

##### Notes

Seifert (2006)

#### Temnothorax strymonensis

Csősz et al. 2018

EF5D0F69-4FCB-5AE8-84F1-AB81A061896F

##### Notes

Csősz et al. (2018), type locality; a Balkan-Anatolian subendemic.

#### Temnothorax tauricus

Ruzsky, 1902

1A41AA01-6D98-5726-BA28-95B2BFBB0505

##### Notes

Radchenko (1994)

#### Temnothorax tergestinus

(Finzi, 1928)

4B9B5F99-9992-522C-BA60-6EFC5F5E6D08

##### Notes

Csősz et al. (2015)

#### Temnothorax tuberum

(Fabricius, 1775)

7189ACD9-BE30-58C5-9DA1-1E4E7A7C09AB

##### Notes

Forel (1892)

#### Temnothorax unifasciatus

(Latreille, 1798)

882AFB50-224C-54F1-BAD0-9C730CBD41FE

##### Notes

Forel (1892)

#### Tetramorium caespitum

(Linnaeus, 1758)

C7E436C1-5FCD-547A-B76F-89421D0A55D0

##### Notes

Forel (1892)

#### Tetramorium cf. punicum

(Smith, 1861)

ABFEACA8-F8F1-527B-A2D0-27586763B5D1

##### Notes

Lapeva-Gjonova and Borowiec (2022)

#### Tetramorium chefketi

Forel, 1911

03632599-357F-5A75-9C34-7D113F5C8622

##### Notes

Atanassov (1952)

#### Tetramorium diomedeum

Emery, 1908

4E75F8BD-9DB0-5B8F-A562-073400F22199

##### Notes

Csősz and Schulz (2010)

#### Tetramorium ferox

Ruzsky, 1903

5C761D57-7FDF-54EB-8D7B-FC9831318C8C

##### Notes

Atanassov and Vasileva (1976)

#### Tetramorium hungaricum

Röszler, 1935

9F16EA6D-B53D-530D-A8B6-F66D0E54900B

##### Notes

Atanassov (1936)

#### Tetramorium immigrans

Santschi, 1927

413C6E03-EEA3-592D-AE76-4637926CB992

##### Notes

Wagner et al. (2017)

#### Tetramorium impurum

(Förster, 1850)

C6A953F0-A022-5900-911C-B9105C5ECED6

##### Notes

Wagner et al. (2017)

#### Tetramorium indocile

Santschi, 1927

A90AB325-B888-5147-A2C6-FD10A49CC69F

##### Notes

Kiran et al. (2017)

#### Tetramorium moravicum

Kratochvil, 1941

4419C9B5-5D55-5E49-8201-23A268967767

##### Notes

Steiner et al. (2005)

#### Tetramorium staerckei

Kratochvíl, 1944

4118423D-9F2B-56E4-9CF6-819646C77DCA

##### Notes

Wagner et al. (2017)

#### Myrmicini

DE816759-6029-5B58-B663-D16312CE9F84

#### Manica rubida

(Latreille, 1802)

6F1A1B73-F2CD-5C3C-B982-4407FF0B3CCC

##### Notes

Forel (1892)

#### Myrmica constricta

Karavaiev, 1934

BAD9F765-0A5E-5BD7-B06F-624209894CBA

##### Notes

Seifert et al. (2009)

#### Myrmica curvithorax

Bondroit, 1920

2FB97AFA-BF1F-509B-AF3F-B5408328CC5A

##### Notes

Sadil (1952)

#### Myrmica gallienii

Bondroit, 1920

49D43D75-A323-537C-8658-DC68F56F61E1

##### Notes

Vassilev (1984)

#### Myrmica hellenica

Finzi, 1926

FB1FE4CA-93DD-541F-9AEA-B5377D136CB8

##### Notes

Seifert (1988b)

#### Myrmica lobicornis

Nylander, 1846

D0ABF35B-C69A-5EC0-8D7C-5E8C3497503A

##### Notes

Forel (1892)

#### Myrmica lonae

Finzi, 1926

18721C15-458D-5E1C-9671-A3104FCF6254

##### Notes

Seifert (2000)

#### Myrmica rubra

(Linnaeus, 1758)

E95AB89F-CC0B-5E14-AD90-9A0398DE23F1

##### Notes

Forel (1892)

#### Myrmica ruginodis

Nylander, 1846

13E3D1B0-EFB4-5B29-A3F5-1ED87051DDF7

##### Notes

Forel (1892)

#### Myrmica rugulosa

Nylander, 1849

C21473B9-50A5-5B94-8657-3FBEEEA271D4

##### Notes

Forel (1892)

#### Myrmica sabuleti

Meinert, 1861

7D2BFB65-7515-5C6B-88BE-499D64F1EFD4

##### Notes

Atanassov (1952)

#### Myrmica scabrinodis

Nylander, 1846

E3A9A637-B298-501D-884B-159BDB4F275D

##### Notes

Forel (1892)

#### Myrmica schencki

Viereck, 1903

799E00F8-8974-58CC-8584-300DF8A06C7E

##### Notes

Atanassov (1952)

#### Myrmica specioides

Bondroit, 1918

E46E8F70-F871-5EFA-9D7E-95AB5513C800

##### Notes

Atanassov and Vasileva (1976)

#### Myrmica sulcinodis

Nylander, 1846

F3AA7239-45B2-5AF4-B9C3-CB795826038C

##### Notes

Forel (1892)

#### Myrmica vandeli

Bondroit, 1920

B27615A8-6A7D-59C8-BD25-54AC5223BA56

##### Notes

Stankiewicz and Antonova (2005)

#### Solenopsidini

AB3061F0-919A-54E7-BC4B-BB448CB58FAF

#### Monomorium monomorium

Bolton, 1987

7635A120-6D03-54F5-ACDB-199338DC80BB

##### Notes

Lapeva-Gjonova and Borowiec (2022)

#### Monomorium pharaonis

(Linnaeus, 1758)

C847AC2A-3B1D-5422-944D-F4F4D94F517A

##### Notes

Atanassov (1965)

#### Solenopsis fugax

(Latreille, 1798)

ADA31F78-2443-5E3F-8E19-F592DBC7EC48

##### Notes

Forel (1892)

#### Stenammini

8086010F-41C4-5FAE-B52E-DBE46D18DE07

#### Aphaenogaster epirotes

(Emery, 1895)

31727F26-F399-598B-BFFA-AF5EEFF19509

##### Notes

Atanassov and Dlusskij (1992)

#### Aphaenogaster festae

Emery, 1915

348E0937-8EA2-53DB-B3F9-5F05A679F0DC

##### Notes

Borowiec et al. (2019); a Balkan-Anatolian subendemic.

#### Aphaenogaster illyrica

Bračko et al., 2019

D574B638-A7EA-5150-9560-998AA4482727

##### Notes

Bračko et al. (2019), paratype locality; a Balkan endemic.

#### Aphaenogaster radchenkoi

Kiran, Aktaç & Tezcan, 2008

BC8E15E7-636E-5AE6-911E-2F1CC8AE22E1

##### Notes

Borowiec et al. (2019); a Balkan-Anatolian subendemic.

#### Aphaenogaster subterranea

(Latreille, 1798)

A27A02D4-437D-54A2-97BF-F44FC37381A0

##### Notes

Forel (1892)

#### Aphaenogaster subterraneoides

Emery, 1881

8779574C-576F-5F13-BC3D-0F6C0746E85B

##### Notes

Borowiec et al. (2019)

#### Messor atanassovii

Atanassov, 1982

63EFF3B1-E616-50D7-93A9-29C97C7BB95B

##### Notes

Atanassov (1982), type locality; a Balkan endemic. Apart from Bulgaria, it is also found in Greek Thrace (L. Borowiec, pers. comm.).

#### Messor hellenius

Agosti & Collingwood, 1987

88730D84-7DF5-57CE-B60A-E02304D0D58C

##### Notes

Lapeva-Gjonova and Borowiec (2022); a Balkan-Anatolian subendemic.

#### Messor ibericus

Santschi, 1925

F6D43941-4E58-572A-BB90-B89A5A9907CC

##### Notes

Steiner et al. (2018)

#### Messor mcarthuri

Steiner et al., 2018

F5DDCEB9-D508-53F6-88ED-7706FD659646

##### Notes

Lapeva-Gjonova and Borowiec (2022); a Balkan-Anatolian subendemic.

#### Messor oertzeni

Forel, 1910

8BDAF5FF-13AF-52D9-8721-D1DBC20C0E24

##### Notes

Atanassov (1934); a Balkan-Anatolian subendemic.

#### Messor ponticus

Steiner et al., 2018

695B2906-0784-5046-A985-C439E3F9278B

##### Notes

Steiner et al. (2018), type locality

#### Messor structor

(Latreille, 1798)

3EC35EE2-32ED-57C5-976F-18F357DC932B

##### Notes

Forel (1892)

#### Messor wasmanni

Krausse, 1910

7ADBE5C3-6B32-5691-8A46-E4FABF572E12

##### Notes

Atanassov (1936)

#### Oxyopomyrmex krueperi

Forel, 1911

7C8D8280-6A20-55AC-8024-7F1129302553

##### Notes

Lapeva-Gjonova and Kiran (2012)

#### Stenamma debile

(Förster, 1850)

B32EE3D4-F3FD-537F-B1A4-DE46E34EE9E4

##### Notes

Emery (1914)

#### Stenamma striatulum

Emery, 1895

CD979007-2446-53B3-BC18-EB4A9A141CBD

##### Notes

Lapeva-Gjonova and Kiran (2012)

#### Ponerinae

AFD1A161-1896-5B3E-BB33-A548C539F864

#### Ponerini

52D1F928-B482-5658-9266-44362B0E993F

#### Cryptopone ochracea

(Mayr, 1855)

37A04783-8BF5-58EC-8919-625E19EC1C9E

##### Notes

Atanassov and Dlusskij (1992)

#### Hypoponera eduardi

(Forel, 1894)

CA6AFD41-3257-5AF5-AC41-994DBF2A138D

##### Notes

Atanassov and Dlusskij (1992)

#### Hypoponera punctatissima

(Roger, 1859)

004A82FC-7DDC-52BF-8162-87E7C86A7152

##### Notes

Atanassov (1936)

#### Ponera coarctata

(Latreille, 1802)

0B6F20B1-CCFF-5770-9BFD-C2BEFF8A8553

##### Notes

Emery (1914)

#### Ponera testacea

Emery, 1895

5AA22E68-C1D5-5C85-981E-488EBA9F5B08

##### Notes

Csősz and Seifert (2003)

#### Proceratiinae

054C0B98-E5B0-509D-A330-24E2B0A6E54C

#### Proceratiini

2F894352-E160-5E04-91F5-39E295949E89

#### Proceratium melinum

(Roger, 1860)

4D47BE8F-A8FB-5A48-9A56-3113210AF7A8

##### Notes

Forel (1895)

#### Proceratium numidicum

Santschi, 1912

EB722EB2-8426-5613-BE4D-4FB5F233A8D6

##### Notes

Agosti and Collingwood (1987)

## Discussion

The current checklist contains 195 species of ants from Bulgaria belonging to six subfamilies and 43 genera. This places Bulgaria amongst the European countries with the highest richness of ant species after Greece (315), Spain (275), Italy (267) and France (215), despite its significantly smaller area (Janicki et al. 2016, Guénard et al. 2017, Salata and Borowiec 2018a,Schifani 2022).

The distribution of species by subfamilies and genera is typical of European myrmecofauna. The richest in genera and species is the subfamily Myrmicinae, containing 23 genera and 106 species, followed by the subfamily Formicinae with 10 genera and 73 species. Thus, the two subfamilies represent 92% of the myrmecofauna in Bulgaria. The most speciose ant genera are *Temnothorax* (Myrmicinae) and *Lasius* (Formicinae) with 27 and 24 species, respectively. More than 10 species are also represented by *Formica* (18), *Camponotus* (16), *Myrmica* (15) and *Tetramorium* (11). Out of all the 43 genera, 26 contain one or two species only.

In this study, records for 24 previously reported species have been re-assessed following taxonomic revisions or reconsideration of available material. The list of excluded species from the current list with remarks and references is given in Table 1.

Due to lack of their exact locality in Bulgaria, four species, namely *Lepisiotanigra* (Dalla Torre, 1893), *Temnothoraxnigriceps* (Mayr, 1855), *Cardiocondylanigra* Forel, 1905 and *Proceratiumnumidicum* Santschi, 1912 (Agosti and Collingwood 1987), seem of doubtful occurrence and their confirmation is needed.

Ant specimens from Bulgaria have been used as holotypes and paratypes for 12 species. Descriptions of three species (*Cardiocondylabulgarica* Forel, 1892, *C.stambuloffii* Forel, 1892 and *Temnothoraxbulgaricus* (Forel, 1892)), still valid today, were already present in the first publication on the ants of Bulgaria from 130 years ago (Forel 1892). Two other species (*Strongylognathusbulgaricus* Pisarski, 1966 and *Teleutomyrmexbuschingeri* Lapeva-Gjonova, 2017) have not yet been reported outside Bulgaria, despite the high probability that they can be found elsewhere in the Balkans.

The Bulgarian myrmecofauna includes 23 endemic and subendemic species, which constitute nearly 12% of all registered ant species in the country. These species are distributed in the two large subfamilies, namely - 17 from Myrmicinae and six from Formicinae. The largest number of species with limited distribution are members of the genera *Messor* (4), *Temnothorax* (4), *Aphaenogaster* (3), *Cardiocondyla* (3), *Camponotus* (3) and *Proformica* (3). Endemics are represented by two species found only in Bulgaria and six species restricted to the Balkans. Both Bulgarian endemics (*Strongylognathusbulgaricus* and *Teleutomyrmexbuschingeri*) are permanent social parasites that are usually extremely rare, although their ant hosts can be common. All six Balkan endemics have records of occurrence only in the southern parts of Bulgaria, where the sub-Mediterranean climatic influence is the strongest. The subendemics are a wider group that includes 14 species distributed over a restricted territory in the Balkan Peninsula and North-West Asia Minor (Balkan-Anatolian species) and one Ponto-Caucasian species.

The presence of rare species and those of great importance for the environment determines the high conservation importance of the ants found in the territory of Bulgaria. In total, 19 ant species have conservation status. Almost all of them (18) are included in the IUCN Red List of Threatened Species (IUCN 2022) and nine are categorised as Vulnerable D2, three as Vulnerable A2c, five as Lower Risk/Near Threatened and one (*Temnothoraxrecedens*) as Lower Risk/Least Concern. The red wood ants are included both in Annex 4 of CORINE biotopes (2000) checklist and in the IUCN Red List (except for *Formicatruncorum*, which is absent from the latter). Additionally, *Formicarufa* is listed in Annexes 2 and 3 of the Bulgarian Biodiversity Act (2002) as protected on the entire Bulgarian territory. The vulnerable species are not currently endangered, but are in a high risk of endangerment in the near future due to threats to natural habitats, declining population, restriction in their area of occupancy or the number of locations. A recently published monitoring of the red wood ants in Bulgaria discussed the status of some of their populations (Antonova and Marinov 2021). However, further research is needed to study in more detail their population dynamics and threats.

An up-to-date assessment of the conservation status of the regional myrmecofauna is needed to reflect both status and taxonomic changes. Thus, potential candidates, such as *Strongylognathusbulgaricus*, *S.huberidalmaticus* and *Teleutomyrmexbuschingeri*, remain off the list for now.

So far, the presence of exotic ant species in Bulgaria is relatively low. These are four species - *Linepithemahumile*, *Lasiusneglectus*, *Monomoriumpharaonis* and *Hypoponerapunctatissima*. All of them are introduced, synanthrope species as *L.humile* and *M.pharaonis* are known only indoors and from greenhouses, while *H.punctatissima* may be found also outdoors in southern parts of Bulgaria (Atanassov and Dlusskij 1992). *Lasiusneglectus* is an invasive urban species, but recently, its colonies have been declining in the country (Tartally et al. 2016).

**Figure 1.**
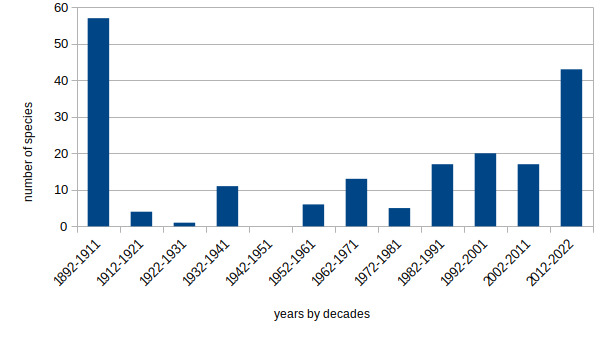
Number of newly-reported ant species from Bulgaria by decades (only currently valid species are considered).

Ant research in Bulgaria dates back to 1892, has continued with variable intensity over the decades and has resulted in 195 species at present (Fig. 1). After Forel's foundational paper with 54 species, more crucial progress in Bulgarian ant research occurred after World War II and with the work of Neno Atanasov. After the 1980s, a number of foreign scientists also contributed to the progress in myrmecological studies. During the last decade, important taxonomic revisions (which included materials from Bulgaria), as well as more intensive research in the southern territories of the country, led to a significant increase in the number of known species in Bulgaria, including descriptions of new ones. However, the number is expected to increase with upcoming surveys and taxonomic revisions.

## Figures and Tables

**Table 1.** Ant species excluded from the list of Bulgaria.

**Excluded species** (by subfamilies)	**Remarks and references**
**Dolichoderinae**	
*Bothriomyrmexgibbus* Soudek, 1925	a junior synonym of *Bothriomyrmexcorsicus* Santschi, 1923 (Seifert 2012)
*Bothriomyrmexmenozzii* Emery, 1925	a junior synonym of *Bothriomyrmexcorsicus* Santschi, 1923 (Seifert 2012)
*Bothriomyrmexmeridionalis* (Roger, 1863)	occurs in Western Europe (France, Spain) (Seifert 2012)
**Formicinae**	
*Camponotussanctus* Forel, 1904	known from Afghanistan, Cyprus, Greece (Aegean Islands, Dodecanese), Iran, Israel, Lebanon, Syria and Turkey (Borowiec and Salata 2020)
*Camponotuspilicornis* (Roger, 1859)	distributed in the Iberian Peninsula and France; the records from Bulgaria are based on misidentification and refer to *Camponotusoertzeni* Forel, 1889 (Lapeva-Gjonova and Borowiec 2022)
*Camponotussylvaticus* (Olivier, 1792)	the records from Bulgaria (Vassilev and Evtimov 1973) probably are based on misidentification (Borowiec 2014)
*Cataglyphislividabulgarica* Atanassov, 1982	a junior synonym of *Cataglyphisviaticoides* (André, 1881) (Salata et al. 2021b)
*Cataglyphisbicolorrufiventris* Emery, 1925	a junior synonym of *Cataglyphisnodus* (Brullé, 1833) (Borowiec and Salata 2013)
*Proformicanasuta* (Nylander, 1856)	a Western Mediterranean species as the records from Bulgaria (Atanassov 1934, Atanassov 1936) are based on misidentifications (Borowiec 2014)
*Plagiolepistaurica* Santschi, 1920	a junior synonym of *Plagiolepispallescens* Forel, 1889 (Salata et al. 2018a)
**Myrmicinae**	
*Aphaenogastergibbosa* (Latreille, 1798)	distributed only in the western part of the Mediterranean Basin (Salata and Borowiec 2018b)
*Aphaenogasterpallida* (Nylander, 1849)	distributed only in the western part of the Mediterranean Basin (Salata and Borowiec 2018b)
*Messorbarbarus* (Linnaeus, 1767)	found only in the Western Palaearctic (Borowiec 2014)
*Messorcaducus* (Motschoulsky, 1839)	restricted to Armenia, Georgia, Iran, Kazakhstan and Turkey (Khalili-Moghadam et al. 2019)
*Messorcapitatus* (Latreille, 1798)	a western Mediterranean species and it is likely that data from the Balkans refer to *M.hellenius* Agosti & Collingwood, 1987 (Salata and Borowiec 2019)
*Messorconcolor* Santschi, 1927	most likely endemic to Crete (Salata and Borowiec 2019
*Crematogasterauberti* Emery, 1869	known from the north-western and western Mediterranean regions; its records from Bulgaria (Lapeva-Gjonova 2011) should be assigned to *C.lorteti* Forel, 1910 (Lapeva-Gjonova and Borowiec 2022)
*Crematogasterscutellaris* (Olivier, 1792)	so far, confirmed findings from the western Mediterranean to the Western Balkans (Croatia) (Borowiec and Salata 2017)
*Temnothoraxmelanocephalus* (Emery, 1870)	a junior synonym of *Temnothoraxtuberum* (Fabricius, 1775) (Casevitz-Weulersse 1990)
*Temnothoraxnylanderi* (Förster, 1850)	known from Central and West Europe: Italy, Austria, Germany and further west; only two species from *Temnothoraxnylanderi* species-complex occur in Bulgaria – *T.crasecundus* Seifert & Csősz 2014 and *T.crassispinus* (Karavaiev, 1926) (Csősz et al. 2015)
*Temnothoraxsaxonicus* (Seifert, 1995)	a junior synonym of *Temnothoraxtergestinus* (Finzi, 1928) Csősz et al. 2015)
*Cardiocondylaelegans* Emery, 1869	a western Mediterranean species; data from the Balkans refer to *Cardiocondyladalmatica* Soudek, 1925 (Seifert 2018)
*Strongylognathuskratochvili* Silhavy, 1937	restricted to Czech Republic and Slovakia; *Strongylognathusbulgaricus* Pisarski, 1966 is revived from synonymy with *S.kratochvili* (Lapeva-Gjonova and Radchenko 2021)
